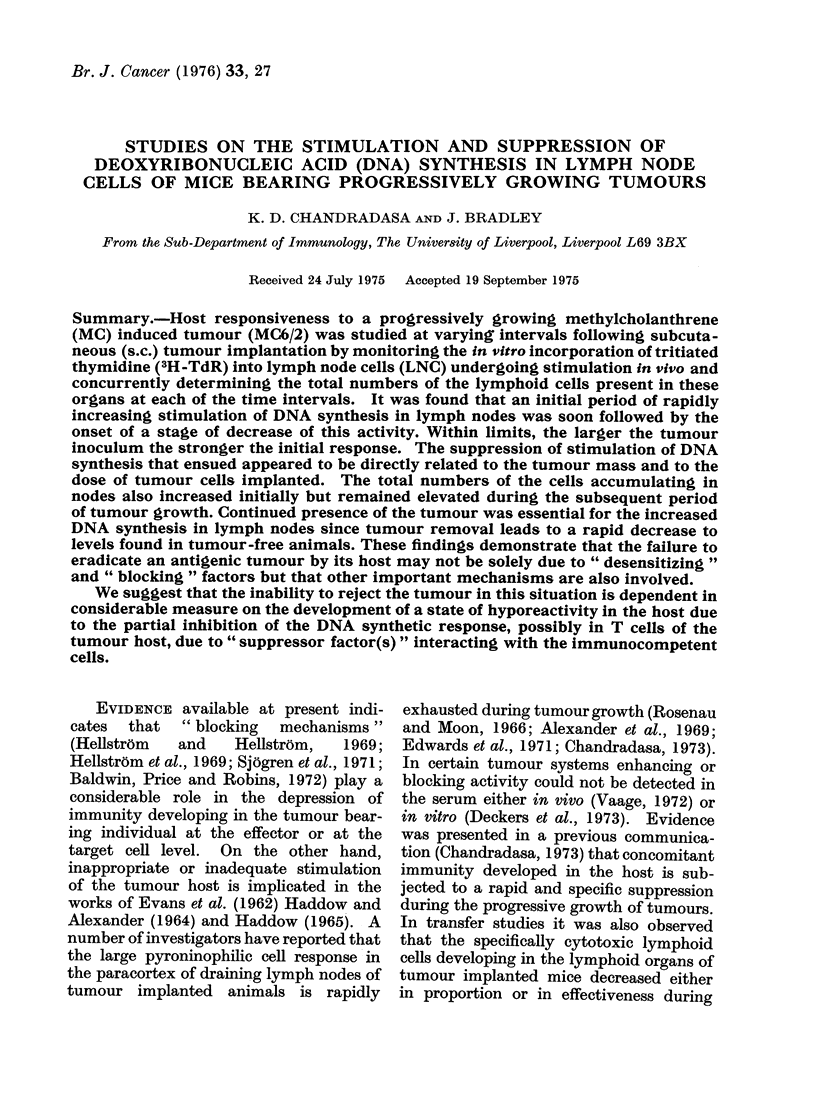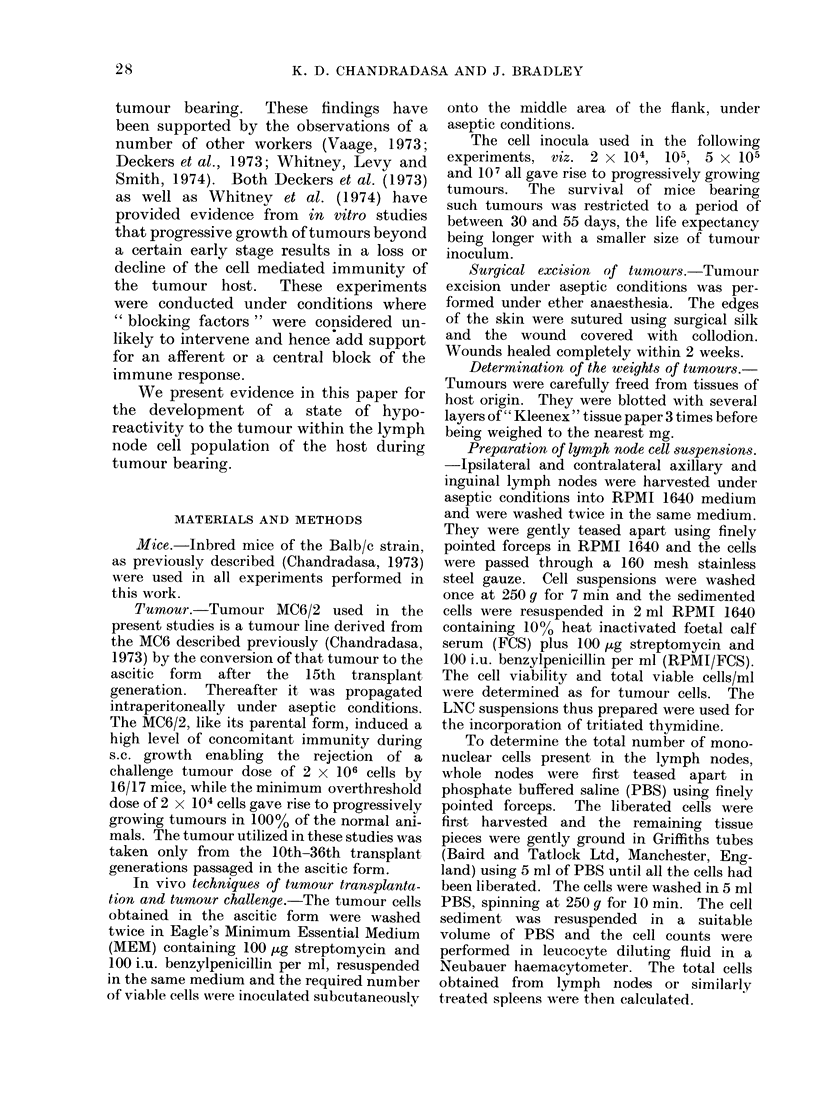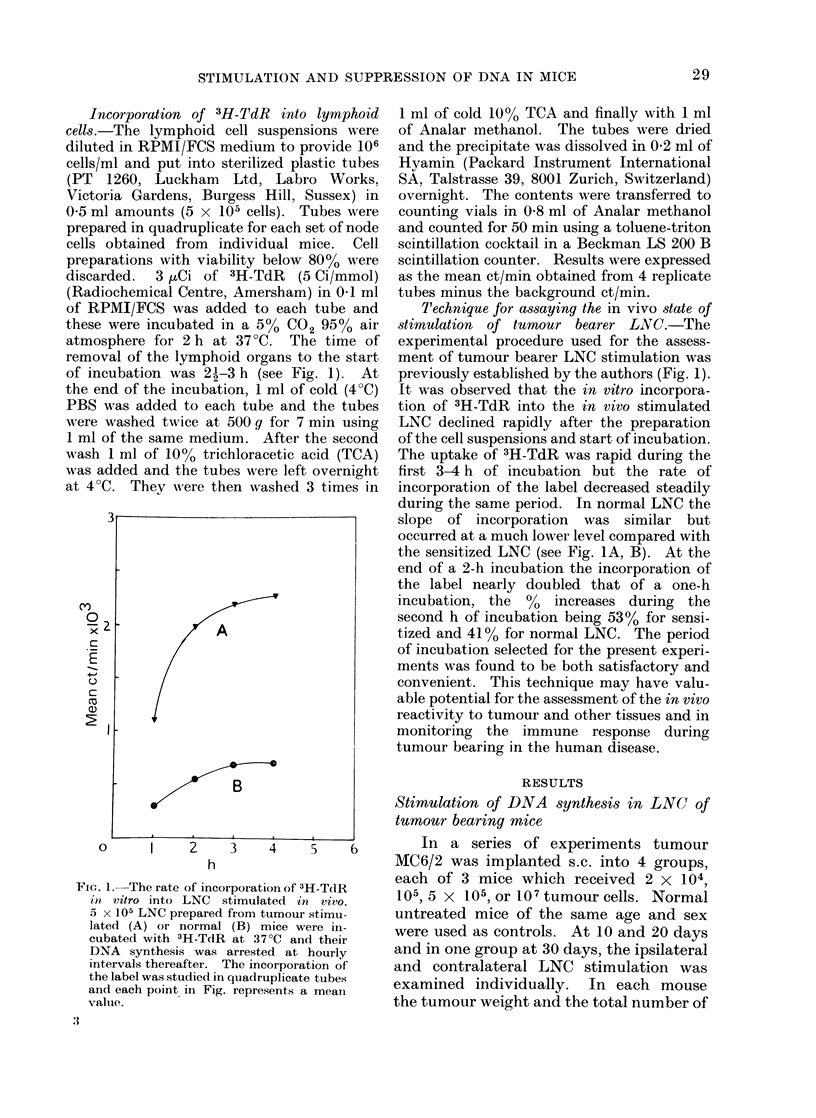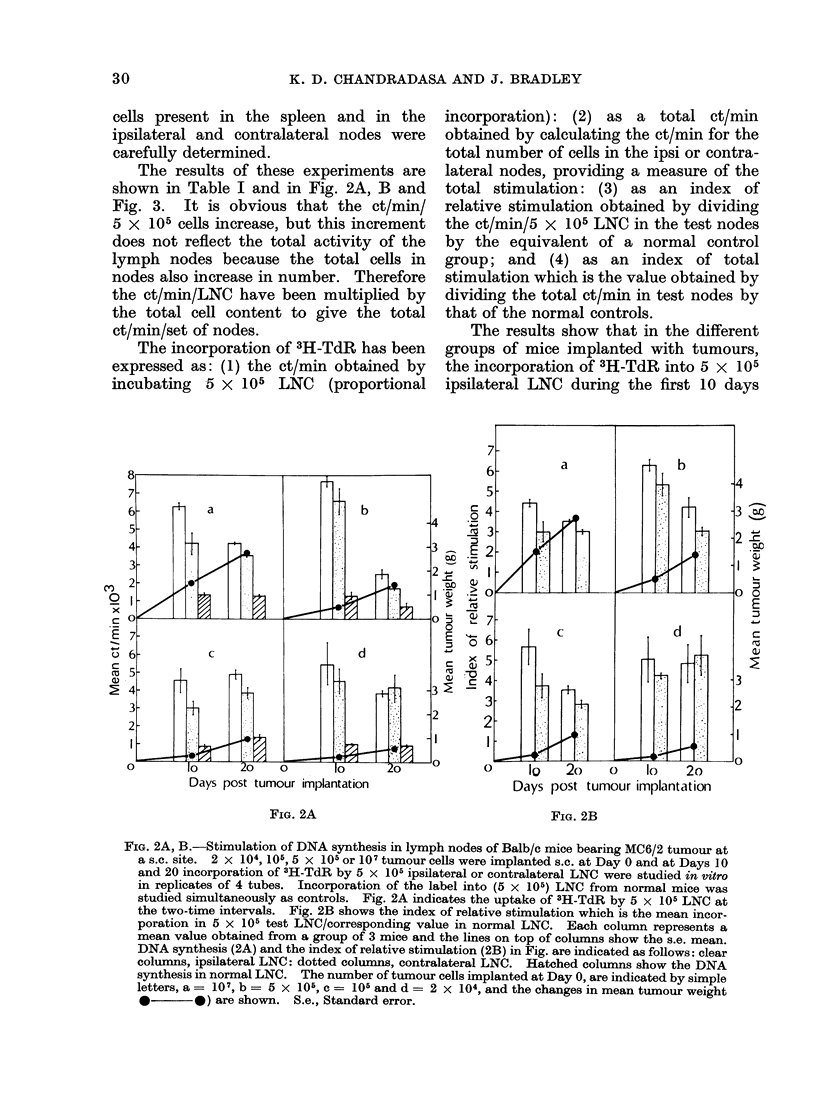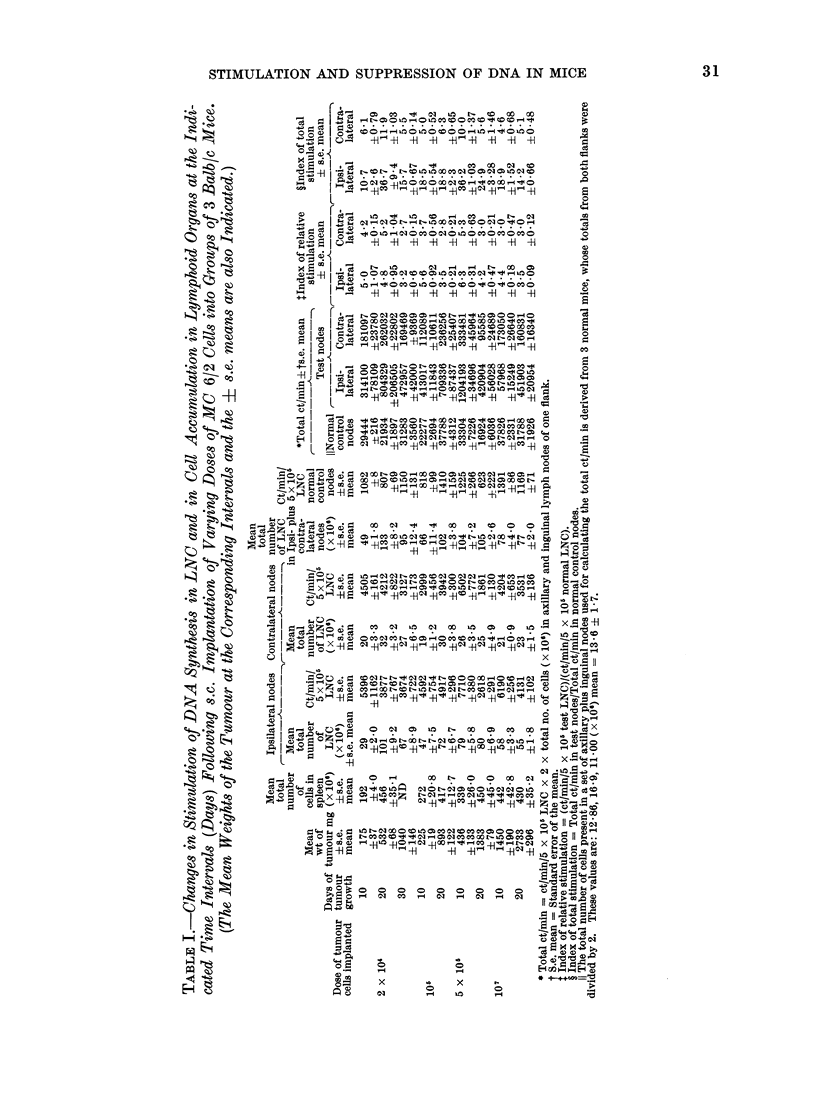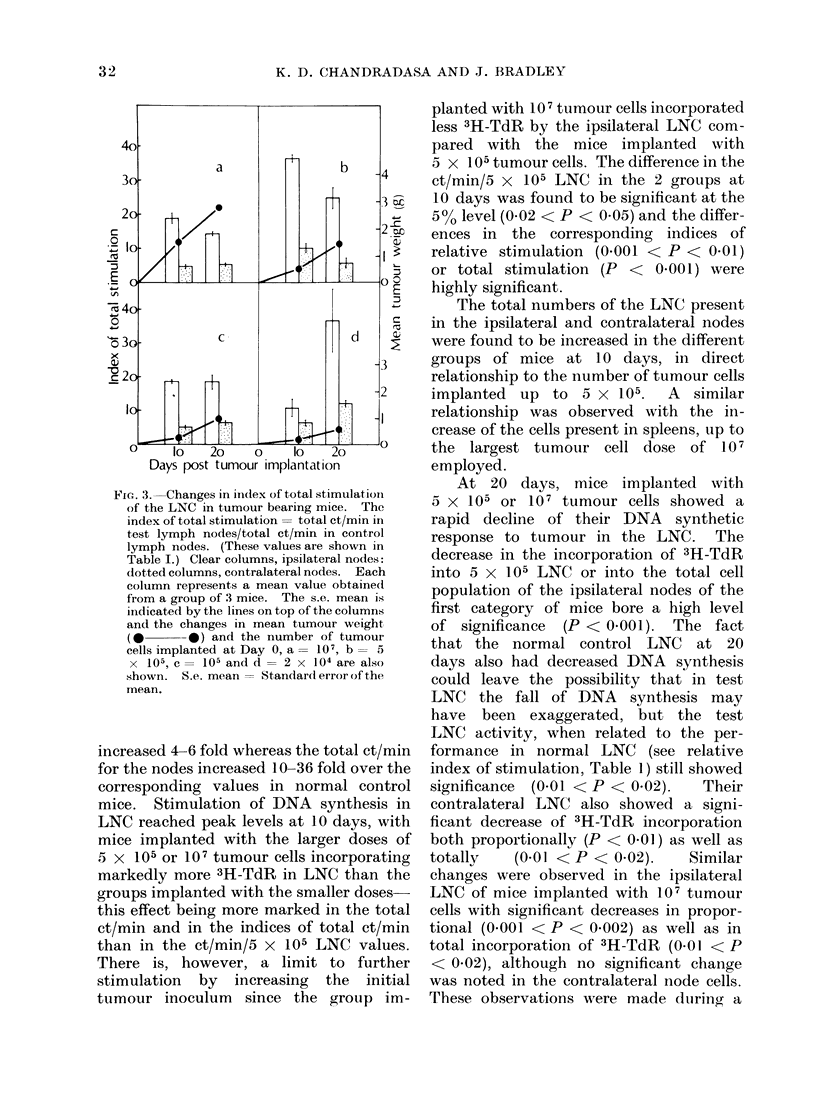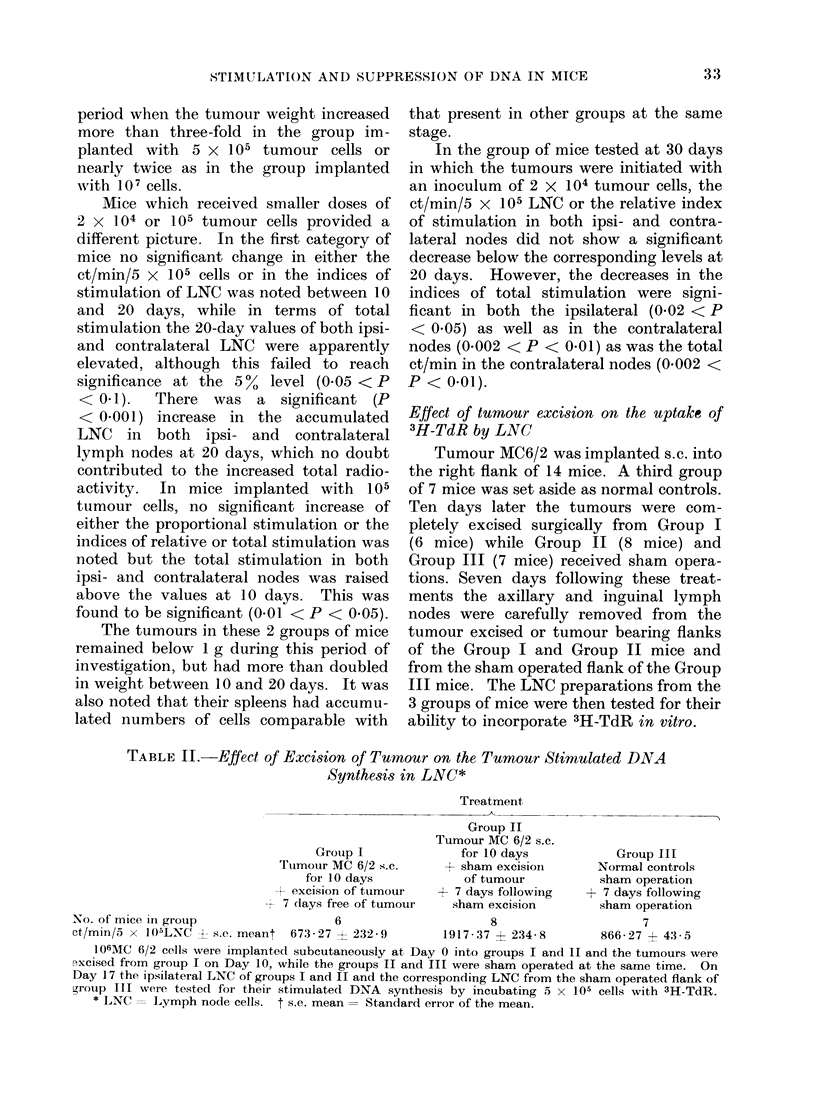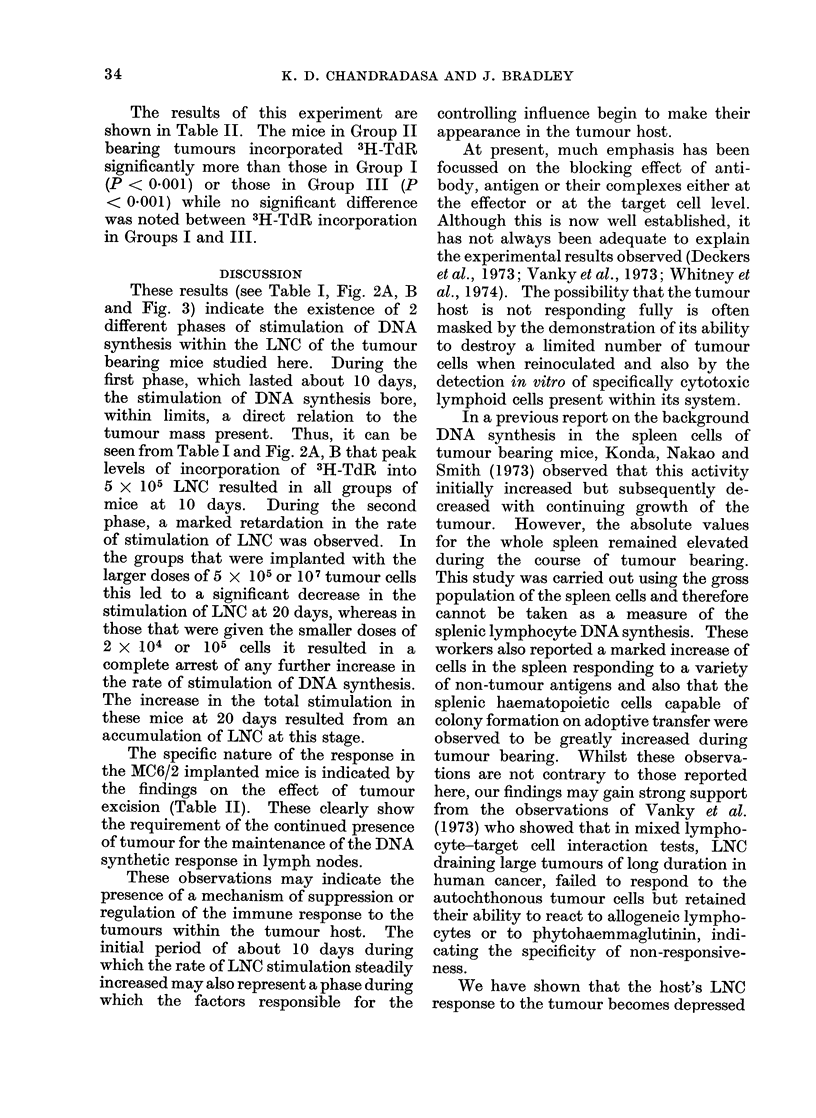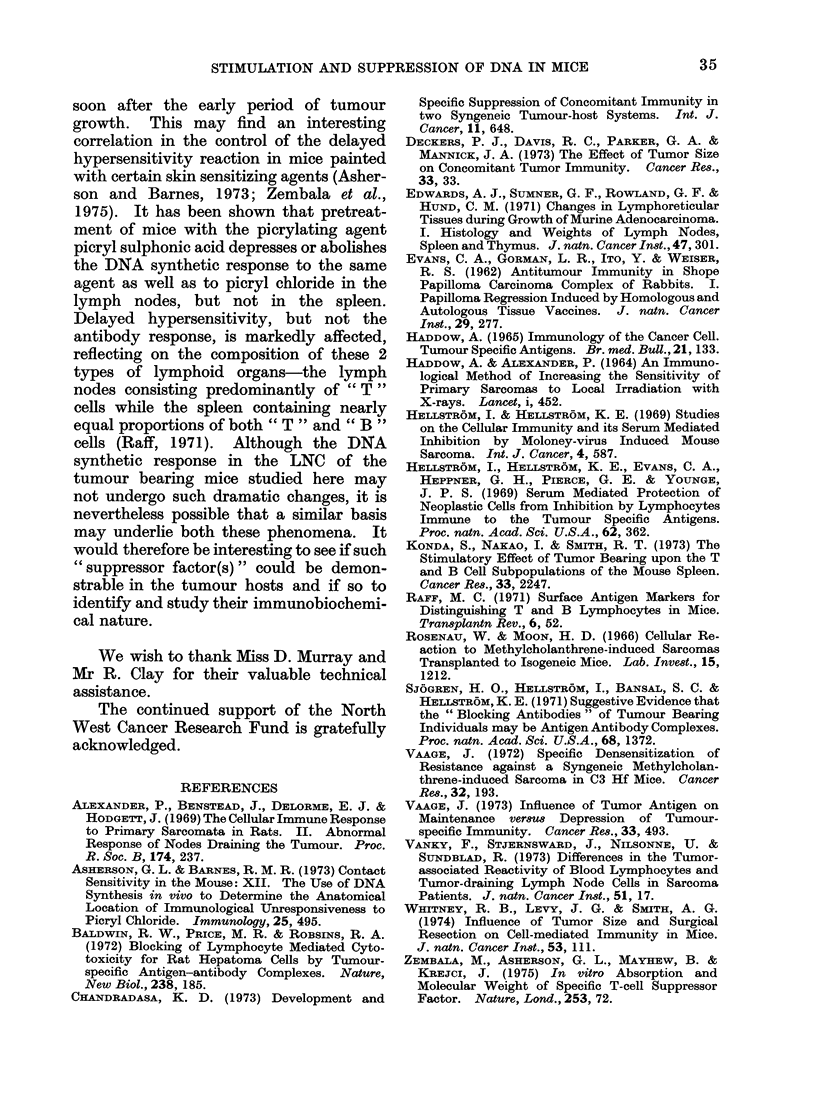# Studies on the stimulation and suppression of deoxyribonucleic acid (DNA) synthesis in lymph node cells of mice bearing progressively growing tumours.

**DOI:** 10.1038/bjc.1976.4

**Published:** 1976-01

**Authors:** K. D. Chandradasa, J. Bradley

## Abstract

Host responsiveness to a progressively growing methylcholanthrene (MC) induced tumour (MC6/2) was studied at varying intervals following subcutaneous (s.c.) tumour implantation by monitoring the in vitro incorporation of tritiated thymidine (3H-TdR) into lymph node cells (LNC) undergoing stimulation in vivo and concurrently determining the total numbers of the lymphoid cells present in these organs at each of the time intervals. It was found that an initial period of rapidly increasing stimulation of DNA synthesis in lymph nodes was soon followed by the onset of a stage of decrease of this activity. Within limits, the larger the tumour inoculum the stronger the initial response. The suppression of stimulation of DNA synthesis that ensued appeared to be directly related to the tumour mass and to the dose of tumour cells implanted. The total numbers of the cells accumulating in nodes also increased initially but remained elevated during the subsequent period of tumour growth. Continued presence of the tumour was essential for the increased DNA synthesis in lymph nodes since tumour removal leads to a rapid decrease to levels found in tumour-free animals. These findings demonstrate that the failure to eradicate an antigenic tumour by its host may not be solely due to "desensitizing" and "blocking" factors but that other important mechanisms are also involved. We suggest that the inability to reject the tumour in this situation is dependent in considerable measure on the development of a state of hyporeactivity in the host due to the partial inhibition of the DNA synthetic response, possibly in T cells of the tumour host, due to "suppressor factor(s)" interacting with the immunocompetent cells.


Br. J. Cancer (1976) 33, 27

### STUDIES ON THE STIMULATION AND SUPPRESSION OF DEOXYRIBONUCLEIC ACID (DNA) SYNTHESIS IN LYMPH NODE CELLS OF MICE BEARING PROGRESSIVELY GROWING TUMOURS

K. D. CHANDRADASA AND J. BRADLEY

*From the Sub-Department of Immunology, The University of Liverpool, Liverpool L69 3BX*

Received 24 July 1975 Accepted 19 September 1975

**Summary.—Host responsiveness to a progressively growing methylcholanthrene (MC) induced tumour (MC6/2) was studied at varying intervals following subcutaneous (s.c.) tumour implantation by monitoring the *in vitro* incorporation of tritiated thymidine ($^3$H-TdR) into lymph node cells (LNC) undergoing stimulation *in vivo* and concurrently determining the total numbers of the lymphoid cells present in these organs at each of the time intervals. It was found that an initial period of rapidly increasing stimulation of DNA synthesis in lymph nodes was soon followed by the onset of a stage of decrease of this activity. Within limits, the larger the tumour inoculum the stronger the initial response. The suppression of stimulation of DNA synthesis that ensued appeared to be directly related to the tumour mass and to the dose of tumour cells implanted. The total numbers of the cells accumulating in nodes also increased initially but remained elevated during the subsequent period of tumour growth. Continued presence of the tumour was essential for the increased DNA synthesis in lymph nodes since tumour removal leads to a rapid decrease to levels found in tumour-free animals. These findings demonstrate that the failure to eradicate an antigenic tumour by its host may not be solely due to " desensitizing " and " blocking " factors but that other important mechanisms are also involved.**

**We suggest that the inability to reject the tumour in this situation is dependent in considerable measure on the development of a state of hyporeactivity in the host due to the partial inhibition of the DNA synthetic response, possibly in T cells of the tumour host, due to " suppressor factor(s) " interacting with the immunocompetent cells.**

EVIDENCE available at present indicates that " blocking mechanisms " (Hellström and Hellström, 1969; Hellström *et al.*, 1969; Sjögren *et al.*, 1971; Baldwin, Price and Robins, 1972) play a considerable role in the depression of immunity developing in the tumour bearing individual at the effector or at the target cell level. On the other hand, inappropriate or inadequate stimulation of the tumour host is implicated in the works of Evans *et al.* (1962) Haddow and Alexander (1964) and Haddow (1965). A number of investigators have reported that the large pyroninophilic cell response in the paracortex of draining lymph nodes of tumour implanted animals is rapidly exhausted during tumour growth (Rosenau and Moon, 1966; Alexander *et al.*, 1969; Edwards *et al.*, 1971; Chandradasa, 1973). In certain tumour systems enhancing or blocking activity could not be detected in the serum either *in vivo* (Vaage, 1972) or *in vitro* (Deckers *et al.*, 1973). Evidence was presented in a previous communication (Chandradasa, 1973) that concomitant immunity developed in the host is subjected to a rapid and specific suppression during the progressive growth of tumours. In transfer studies it was also observed that the specifically cytotoxic lymphoid cells developing in the lymphoid organs of tumour implanted mice decreased either in proportion or in effectiveness during tumour bearing. These findings have been supported by the observations of a number of other workers (Vaage, 1973; Deckers *et al.*, 1973; Whitney, Levy and Smith, 1974). Both Deckers *et al.* (1973) as well as Whitney *et al.* (1974) have provided evidence from *in vitro* studies that progressive growth of tumours beyond a certain early stage results in a loss or decline of the cell mediated immunity of the tumour host. These experiments were conducted under conditions where " blocking factors " were considered unlikely to intervene and hence add support for an afferent or a central block of the immune response.

We present evidence in this paper for the development of a state of hyporeactivity to the tumour within the lymph node cell population of the host during tumour bearing.

### MATERIALS AND METHODS

*Mice.*—Inbred mice of the Balb/c strain, as previously described (Chandradasa, 1973) were used in all experiments performed in this work.

*Tumour.*—Tumour MC6/2 used in the present studies is a tumour line derived from the MC6 described previously (Chandradasa, 1973) by the conversion of that tumour to the ascitic form after the 15th transplant generation. Thereafter it was propagated intraperitoneally under aseptic conditions. The MC6/2, like its parental form, induced a high level of concomitant immunity during s.c. growth enabling the rejection of a challenge tumour dose of $2 \times 10^6$ cells by 16/17 mice, while the minimum overthreshold dose of $2 \times 10^4$ cells gave rise to progressively growing tumours in 100% of the normal animals. The tumour utilized in these studies was taken only from the 10th–36th transplant generations passaged in the ascitic form.

In vivo *techniques of tumour transplantation and tumour challenge.*—The tumour cells obtained in the ascitic form were washed twice in Eagle's Minimum Essential Medium (MEM) containing 100 $\mu$g streptomycin and 100 i.u. benzylpenicillin per ml, resuspended in the same medium and the required number of viable cells were inoculated subcutaneously onto the middle area of the flank, under aseptic conditions.

The cell inocula used in the following experiments, *viz.* $2 \times 10^4$, $10^5$, $5 \times 10^5$ and $10^7$ all gave rise to progressively growing tumours. The survival of mice bearing such tumours was restricted to a period of between 30 and 55 days, the life expectancy being longer with a smaller size of tumour inoculum.

*Surgical excision of tumours.*—Tumour excision under aseptic conditions was performed under ether anaesthesia. The edges of the skin were sutured using surgical silk and the wound covered with collodion. Wounds healed completely within 2 weeks.

*Determination of the weights of tumours.*—Tumours were carefully freed from tissues of host origin. They were blotted with several layers of "Kleenex" tissue paper 3 times before being weighed to the nearest mg.

*Preparation of lymph node cell suspensions.*—Ipsilateral and contralateral axillary and inguinal lymph nodes were harvested under aseptic conditions into RPMI 1640 medium and were washed twice in the same medium. They were gently teased apart using finely pointed forceps in RPMI 1640 and the cells were passed through a 160 mesh stainless steel gauze. Cell suspensions were washed once at 250 *g* for 7 min and the sedimented cells were resuspended in 2 ml RPMI 1640 containing 10% heat inactivated foetal calf serum (FCS) plus 100 $\mu$g streptomycin and 100 i.u. benzylpenicillin per ml (RPMI/FCS). The cell viability and total viable cells/ml were determined as for tumour cells. The LNC suspensions thus prepared were used for the incorporation of tritiated thymidine.

To determine the total number of mononuclear cells present in the lymph nodes, whole nodes were first teased apart in phosphate buffered saline (PBS) using finely pointed forceps. The liberated cells were first harvested and the remaining tissue pieces were gently ground in Griffiths tubes (Baird and Tatlock Ltd, Manchester, England) using 5 ml of PBS until all the cells had been liberated. The cells were washed in 5 ml PBS, spinning at 250 *g* for 10 min. The cell sediment was resuspended in a suitable volume of PBS and the cell counts were performed in leucocyte diluting fluid in a Neubauer haemacytometer. The total cells obtained from lymph nodes or similarly treated spleens were then calculated.

*Incorporation of $^3$H-TdR into lymphoid cells.*—The lymphoid cell suspensions were diluted in RPMI/FCS medium to provide $10^6$ cells/ml and put into sterilized plastic tubes (PT 1260, Luckham Ltd, Labro Works, Victoria Gardens, Burgess Hill, Sussex) in 0·5 ml amounts ($5 \times 10^5$ cells). Tubes were prepared in quadruplicate for each set of node cells obtained from individual mice. Cell preparations with viability below 80% were discarded. 3 $\mu$Ci of $^3$H-TdR (5 Ci/mmol) (Radiochemical Centre, Amersham) in 0·1 ml of RPMI/FCS was added to each tube and these were incubated in a 5% $CO_2$ 95% air atmosphere for 2 h at 37°C. The time of removal of the lymphoid organs to the start of incubation was 2½–3 h (see Fig. 1). At the end of the incubation, 1 ml of cold (4°C) PBS was added to each tube and the tubes were washed twice at 500 *g* for 7 min using 1 ml of the same medium. After the second wash 1 ml of 10% trichloracetic acid (TCA) was added and the tubes were left overnight at 4°C. They were then washed 3 times in 1 ml of cold 10% TCA and finally with 1 ml of Analar methanol. The tubes were dried and the precipitate was dissolved in 0·2 ml of Hyamin (Packard Instrument International SA, Talstrasse 39, 8001 Zurich, Switzerland) overnight. The contents were transferred to counting vials in 0·8 ml of Analar methanol and counted for 50 min using a toluene-triton scintillation cocktail in a Beckman LS 200 B scintillation counter. Results were expressed as the mean ct/min obtained from 4 replicate tubes minus the background ct/min.

FIG. 1.—The rate of incorporation of $^3$H-TdR *in vitro* into LNC stimulated *in vivo*. $5 \times 10^5$ LNC prepared from tumour stimulated (A) or normal (B) mice were incubated with $^3$H-TdR at 37°C and their DNA synthesis was arrested at hourly intervals thereafter. The incorporation of the label was studied in quadruplicate tubes and each point in Fig. represents a mean value.

*Technique for assaying the* in vivo *state of stimulation of tumour bearer LNC.*—The experimental procedure used for the assessment of tumour bearer LNC stimulation was previously established by the authors (Fig. 1). It was observed that the *in vitro* incorporation of $^3$H-TdR into the *in vivo* stimulated LNC declined rapidly after the preparation of the cell suspensions and start of incubation. The uptake of $^3$H-TdR was rapid during the first 3–4 h of incubation but the rate of incorporation of the label decreased steadily during the same period. In normal LNC the slope of incorporation was similar but occurred at a much lower level compared with the sensitized LNC (see Fig. 1A, B). At the end of a 2-h incubation the incorporation of the label nearly doubled that of a one-h incubation, the % increases during the second h of incubation being 53% for sensitized and 41% for normal LNC. The period of incubation selected for the present experiments was found to be both satisfactory and convenient. This technique may have valuable potential for the assessment of the *in vivo* reactivity to tumour and other tissues and in monitoring the immune response during tumour bearing in the human disease.

### RESULTS

### *Stimulation of DNA synthesis in LNC of tumour bearing mice*

In a series of experiments tumour MC6/2 was implanted s.c. into 4 groups, each of 3 mice which received $2 \times 10^4$, $10^5$, $5 \times 10^5$, or $10^7$ tumour cells. Normal untreated mice of the same age and sex were used as controls. At 10 and 20 days and in one group at 30 days, the ipsilateral and contralateral LNC stimulation was examined individually. In each mouse the tumour weight and the total number of cells present in the spleen and in the ipsilateral and contralateral nodes were carefully determined.

The results of these experiments are shown in Table I and in Fig. 2A, B and Fig. 3. It is obvious that the ct/min/ $5 \times 10^5$ cells increase, but this increment does not reflect the total activity of the lymph nodes because the total cells in nodes also increase in number. Therefore the ct/min/LNC have been multiplied by the total cell content to give the total ct/min/set of nodes.

The incorporation of $^3$H-TdR has been expressed as: (1) the ct/min obtained by incubating $5 \times 10^5$ LNC (proportional incorporation): (2) as a total ct/min obtained by calculating the ct/min for the total number of cells in the ipsi or contralateral nodes, providing a measure of the total stimulation: (3) as an index of relative stimulation obtained by dividing the ct/min/$5 \times 10^5$ LNC in the test nodes by the equivalent of a normal control group; and (4) as an index of total stimulation which is the value obtained by dividing the total ct/min in test nodes by that of the normal controls.

The results show that in the different groups of mice implanted with tumours, the incorporation of $^3$H-TdR into $5 \times 10^5$ ipsilateral LNC during the first 10 days

Fig. 2A

Fig. 2B

Fig. 2A, B.—Stimulation of DNA synthesis in lymph nodes of Balb/c mice bearing MC6/2 tumour at a s.c. site. $2 \times 10^4$, $10^5$, $5 \times 10^5$ or $10^7$ tumour cells were implanted s.c. at Day 0 and at Days 10 and 20 incorporation of $^3$H-TdR by $5 \times 10^5$ ipsilateral or contralateral LNC were studied *in vitro* in replicates of 4 tubes. Incorporation of the label into ($5 \times 10^5$) LNC from normal mice was studied simultaneously as controls. Fig. 2A indicates the uptake of $^3$H-TdR by $5 \times 10^5$ LNC at the two-time intervals. Fig. 2B shows the index of relative stimulation which is the mean incorporation in $5 \times 10^5$ test LNC/corresponding value in normal LNC. Each column represents a mean value obtained from a group of 3 mice and the lines on top of columns show the s.e. mean. DNA synthesis (2A) and the index of relative stimulation (2B) in Fig. are indicated as follows: clear columns, ipsilateral LNC: dotted columns, contralateral LNC. Hatched columns show the DNA synthesis in normal LNC. The number of tumour cells implanted at Day 0, are indicated by simple letters, a = $10^7$, b = $5 \times 10^5$, c = $10^5$ and d = $2 \times 10^4$, and the changes in mean tumour weight (●——●) are shown. S.e., Standard error.

TABLE I.—*Changes in Stimulation of DNA Synthesis in LNC and in Cell Accumulation in Lymphoid Organs at the Indicated Time Intervals (Days) Following s.c. Implantation of Varying Doses of MC 6/2 Cells into Groups of 3 Balb/c Mice. (The Mean Weights of the Tumour at the Corresponding Intervals and the ± s.e. means are also Indicated.)*

| Dose of tumour cells implanted | Days of tumour growth | Mean wt of tumour mg ±s.e. mean | Mean total number of cells in spleen ($\times 10^6$) ±s.e. mean | Ipsilateral nodes: Mean total number of LNC ($\times 10^6$) ±s.e. mean | Ipsilateral nodes: Ct/min/ $5\times10^5$ LNC ±s.e. mean | Contralateral nodes: Mean total number of LNC ($\times 10^6$) ±s.e. mean | Contralateral nodes: Ct/min/ $5\times10^5$ LNC ±s.e. mean | Mean total number of LNC in Ipsi- plus contra-lateral nodes ($\times 10^6$) ±s.e. mean | Ct/min/ $5\times10^5$ LNC normal control nodes ±s.e. mean | *Total ct/min ± †s.e. mean: ‖Normal control nodes | *Total ct/min ± †s.e. mean: Test nodes Ipsi-lateral | *Total ct/min ± †s.e. mean: Test nodes Contra-lateral | ‡Index of relative stimulation ± s.e. mean: Ipsi-lateral | ‡Index of relative stimulation ± s.e. mean: Contra-lateral | §Index of total stimulation ± s.e. mean: Ipsi-lateral | §Index of total stimulation ± s.e. mean: Contra-lateral |
|---|---|---|---|---|---|---|---|---|---|---|---|---|---|---|---|---|
| 2 × 10⁴ | 10 | 175 ±37 | 192 ±4·0 | 29 ±2·0 | 5396 ±1162 | 20 ±3·3 | 4505 ±161 | 49 ±1·8 | 1082 ±8 | 29444 ±216 | 314100 ±78109 | 181097 ±23780 | 5·0 ±1·07 | 4·2 ±0·15 | 10·7 ±2·6 | 6·1 ±0·79 |
| | 20 | 532 ±68 | 456 ±35·1 | 101 ±9·2 | 3877 ±767 | 32 ±3·2 | 4212 ±822 | 133 ±8·2 | 807 ±69 | 21934 ±1897 | 804329 ±206505 | 262032 ±22802 | 4·8 ±0·95 | 5·2 ±1·04 | 36·7 ±9·4 | 11·9 ±1·03 |
| | 30 | 1040 ±146 | ND | 67 ±8·9 | 3674 ±722 | 27 ±6·5 | 3127 ±173 | 95 ±12·4 | 1150 ±131 | 31283 ±3560 | 472957 ±42000 | 169469 ±9369 | 3·2 ±0·6 | 2·7 ±0·15 | 15·7 ±0·67 | 5·5 ±0·14 |
| 10⁵ | 10 | 225 ±19 | 272 ±20·8 | 47 ±7·5 | 4592 ±754 | 19 ±1·2 | 2999 ±456 | 66 ±11·4 | 818 ±99 | 22277 ±2694 | 413017 ±11843 | 112089 ±10611 | 5·6 ±0·92 | 3·7 ±0·56 | 18·5 ±0·54 | 5·0 ±0·52 |
| | 20 | 893 ±122 | 417 ±12·7 | 72 ±6·7 | 4917 ±296 | 30 ±3·8 | 3942 ±300 | 102 ±3·8 | 1410 ±159 | 37788 ±4312 | 709336 ±87437 | 236256 ±25407 | 3·5 ±0·21 | 2·8 ±0·21 | 18·8 ±2·3 | 6·3 ±0·65 |
| 5 × 10⁵ | 10 | 436 ±133 | 339 ±26·0 | 79 ±5·8 | 7710 ±380 | 26 ±3·5 | 6502 ±772 | 104 ±7·2 | 1225 ±266 | 33304 ±7226 | 1204193 ±34696 | 333481 ±45964 | 6·3 ±0·31 | 5·3 ±0·63 | 36·2 ±1·03 | 10·0 ±1·37 |
| | 20 | 1383 ±79 | 450 ±45·0 | 80 ±6·9 | 2618 ±291 | 25 ±4·9 | 1861 ±130 | 105 ±2·6 | 623 ±222 | 16924 ±6036 | 420904 ±56028 | 95585 ±24689 | 4·2 ±0·47 | 3·0 ±0·21 | 24·9 ±3·28 | 5·6 ±1·46 |
| 10⁷ | 10 | 1450 ±190 | 442 ±42·8 | 58 ±3·3 | 6190 ±256 | 21 ±0·9 | 4204 ±653 | 78 ±4·0 | 1391 ±86 | 37826 ±2331 | 57968 ±15249 | 173050 ±26640 | 4·4 ±0·18 | 3·0 ±0·47 | 18·9 ±1·52 | 4·6 ±0·68 |
| | 20 | 2733 ±296 | 430 ±35·2 | 55 ±1·8 | 4131 ±102 | 23 ±1·5 | 3531 ±136 | 77 ±2·0 | 1169 ±71 | 31788 ±1926 | 451903 ±20954 | 160831 ±16340 | 3·5 ±0·09 | 3·0 ±0·12 | 14·2 ±0·66 | 5·1 ±0·48 |

* Total ct/min = ct/min/5 × 10⁵ LNC × 2 × total no. of cells (× 10⁶) in axillary and inguinal lymph nodes of one flank.

† S.e. mean = Standard error of the mean.

‡ Index of relative stimulation = (ct/min/5 × 10⁵ test LNC)/(ct/min/5 × 10⁵ normal LNC).

§ Index of total stimulation = Total ct/min in test nodes/Total ct/min in normal control nodes.

‖ The total number of cells present in a set of axillary plus inguinal nodes used for calculating the total ct/min is derived from 3 normal mice, whose totals from both flanks were divided by 2. These values are: 12·86, 16·9, 11·00 (× 10⁶) mean = 13·6 ± 1·7.

FIG. 3.—Changes in index of total stimulation of the LNC in tumour bearing mice. The index of total stimulation = total ct/min in test lymph nodes/total ct/min in control lymph nodes. (These values are shown in Table I.) Clear columns, ipsilateral nodes: dotted columns, contralateral nodes. Each column represents a mean value obtained from a group of 3 mice. The s.e. mean is indicated by the lines on top of the columns and the changes in mean tumour weight (●———●) and the number of tumour cells implanted at Day 0, a = $10^7$, b = $5 \times 10^5$, c = $10^5$ and d = $2 \times 10^4$ are also shown. S.e. mean = Standard error of the mean.

increased 4–6 fold whereas the total ct/min for the nodes increased 10–36 fold over the corresponding values in normal control mice. Stimulation of DNA synthesis in LNC reached peak levels at 10 days, with mice implanted with the larger doses of $5 \times 10^5$ or $10^7$ tumour cells incorporating markedly more $^3$H-TdR in LNC than the groups implanted with the smaller doses—this effect being more marked in the total ct/min and in the indices of total ct/min than in the ct/min/$5 \times 10^5$ LNC values. There is, however, a limit to further stimulation by increasing the initial tumour inoculum since the group implanted with $10^7$ tumour cells incorporated less $^3$H-TdR by the ipsilateral LNC compared with the mice implanted with $5 \times 10^5$ tumour cells. The difference in the ct/min/$5 \times 10^5$ LNC in the 2 groups at 10 days was found to be significant at the 5% level ($0{\cdot}02 < P < 0{\cdot}05$) and the differences in the corresponding indices of relative stimulation ($0{\cdot}001 < P < 0{\cdot}01$) or total stimulation ($P < 0{\cdot}001$) were highly significant.

The total numbers of the LNC present in the ipsilateral and contralateral nodes were found to be increased in the different groups of mice at 10 days, in direct relationship to the number of tumour cells implanted up to $5 \times 10^5$. A similar relationship was observed with the increase of the cells present in spleens, up to the largest tumour cell dose of $10^7$ employed.

At 20 days, mice implanted with $5 \times 10^5$ or $10^7$ tumour cells showed a rapid decline of their DNA synthetic response to tumour in the LNC. The decrease in the incorporation of $^3$H-TdR into $5 \times 10^5$ LNC or into the total cell population of the ipsilateral nodes of the first category of mice bore a high level of significance ($P < 0{\cdot}001$). The fact that the normal control LNC at 20 days also had decreased DNA synthesis could leave the possibility that in test LNC the fall of DNA synthesis may have been exaggerated, but the test LNC activity, when related to the performance in normal LNC (see relative index of stimulation, Table 1) still showed significance ($0{\cdot}01 < P < 0{\cdot}02$). Their contralateral LNC also showed a significant decrease of $^3$H-TdR incorporation both proportionally ($P < 0{\cdot}01$) as well as totally ($0{\cdot}01 < P < 0{\cdot}02$). Similar changes were observed in the ipsilateral LNC of mice implanted with $10^7$ tumour cells with significant decreases in proportional ($0{\cdot}001 < P < 0{\cdot}002$) as well as in total incorporation of $^3$H-TdR ($0{\cdot}01 < P < 0{\cdot}02$), although no significant change was noted in the contralateral node cells. These observations were made during a period when the tumour weight increased more than three-fold in the group implanted with $5 \times 10^5$ tumour cells or nearly twice as in the group implanted with $10^7$ cells.

Mice which received smaller doses of $2 \times 10^4$ or $10^5$ tumour cells provided a different picture. In the first category of mice no significant change in either the ct/min/$5 \times 10^5$ cells or in the indices of stimulation of LNC was noted between 10 and 20 days, while in terms of total stimulation the 20-day values of both ipsi- and contralateral LNC were apparently elevated, although this failed to reach significance at the 5% level ($0{\cdot}05 < P < 0{\cdot}1$). There was a significant ($P < 0{\cdot}001$) increase in the accumulated LNC in both ipsi- and contralateral lymph nodes at 20 days, which no doubt contributed to the increased total radioactivity. In mice implanted with $10^5$ tumour cells, no significant increase of either the proportional stimulation or the indices of relative or total stimulation was noted but the total stimulation in both ipsi- and contralateral nodes was raised above the values at 10 days. This was found to be significant ($0{\cdot}01 < P < 0{\cdot}05$).

The tumours in these 2 groups of mice remained below 1 g during this period of investigation, but had more than doubled in weight between 10 and 20 days. It was also noted that their spleens had accumulated numbers of cells comparable with that present in other groups at the same stage.

In the group of mice tested at 30 days in which the tumours were initiated with an inoculum of $2 \times 10^4$ tumour cells, the ct/min/$5 \times 10^5$ LNC or the relative index of stimulation in both ipsi- and contralateral nodes did not show a significant decrease below the corresponding levels at 20 days. However, the decreases in the indices of total stimulation were significant in both the ipsilateral ($0{\cdot}02 < P < 0{\cdot}05$) as well as in the contralateral nodes ($0{\cdot}002 < P < 0{\cdot}01$) as was the total ct/min in the contralateral nodes ($0{\cdot}002 < P < 0{\cdot}01$).

*Effect of tumour excision on the uptake of $^3$H-TdR by LNC*

Tumour MC6/2 was implanted s.c. into the right flank of 14 mice. A third group of 7 mice was set aside as normal controls. Ten days later the tumours were completely excised surgically from Group I (6 mice) while Group II (8 mice) and Group III (7 mice) received sham operations. Seven days following these treatments the axillary and inguinal lymph nodes were carefully removed from the tumour excised or tumour bearing flanks of the Group I and Group II mice and from the sham operated flank of the Group III mice. The LNC preparations from the 3 groups of mice were then tested for their ability to incorporate $^3$H-TdR *in vitro*.

TABLE II.—*Effect of Excision of Tumour on the Tumour Stimulated DNA Synthesis in LNC**

| | Treatment | | |
|---|---|---|---|
| | Group I Tumour MC 6/2 s.c. for 10 days + excision of tumour + 7 days free of tumour | Group II Tumour MC 6/2 s.c. for 10 days + sham excision of tumour + 7 days following sham excision | Group III Normal controls sham operation + 7 days following sham operation |
| No. of mice in group | 6 | 8 | 7 |
| ct/min/$5 \times 10^5$LNC ± s.e. mean† | $673{\cdot}27 \pm 232{\cdot}9$ | $1917{\cdot}37 \pm 234{\cdot}8$ | $866{\cdot}27 \pm 43{\cdot}5$ |

$10^6$MC 6/2 cells were implanted subcutaneously at Day 0 into groups I and II and the tumours were excised from group I on Day 10, while the groups II and III were sham operated at the same time. On Day 17 the ipsilateral LNC of groups I and II and the corresponding LNC from the sham operated flank of group III were tested for their stimulated DNA synthesis by incubating $5 \times 10^5$ cells with $^3$H-TdR.

* LNC = Lymph node cells. † s.e. mean = Standard error of the mean.

The results of this experiment are shown in Table II. The mice in Group II bearing tumours incorporated $^3$H-TdR significantly more than those in Group I ($P < 0{\cdot}001$) or those in Group III ($P < 0{\cdot}001$) while no significant difference was noted between $^3$H-TdR incorporation in Groups I and III.

### DISCUSSION

These results (see Table I, Fig. 2A, B and Fig. 3) indicate the existence of 2 different phases of stimulation of DNA synthesis within the LNC of the tumour bearing mice studied here. During the first phase, which lasted about 10 days, the stimulation of DNA synthesis bore, within limits, a direct relation to the tumour mass present. Thus, it can be seen from Table I and Fig. 2A, B that peak levels of incorporation of $^3$H-TdR into $5 \times 10^5$ LNC resulted in all groups of mice at 10 days. During the second phase, a marked retardation in the rate of stimulation of LNC was observed. In the groups that were implanted with the larger doses of $5 \times 10^5$ or $10^7$ tumour cells this led to a significant decrease in the stimulation of LNC at 20 days, whereas in those that were given the smaller doses of $2 \times 10^4$ or $10^5$ cells it resulted in a complete arrest of any further increase in the rate of stimulation of DNA synthesis. The increase in the total stimulation in these mice at 20 days resulted from an accumulation of LNC at this stage.

The specific nature of the response in the MC6/2 implanted mice is indicated by the findings on the effect of tumour excision (Table II). These clearly show the requirement of the continued presence of tumour for the maintenance of the DNA synthetic response in lymph nodes.

These observations may indicate the presence of a mechanism of suppression or regulation of the immune response to the tumours within the tumour host. The initial period of about 10 days during which the rate of LNC stimulation steadily increased may also represent a phase during which the factors responsible for the controlling influence begin to make their appearance in the tumour host.

At present, much emphasis has been focussed on the blocking effect of antibody, antigen or their complexes either at the effector or at the target cell level. Although this is now well established, it has not always been adequate to explain the experimental results observed (Deckers *et al.*, 1973; Vanky *et al.*, 1973; Whitney *et al.*, 1974). The possibility that the tumour host is not responding fully is often masked by the demonstration of its ability to destroy a limited number of tumour cells when reinoculated and also by the detection *in vitro* of specifically cytotoxic lymphoid cells present within its system.

In a previous report on the background DNA synthesis in the spleen cells of tumour bearing mice, Konda, Nakao and Smith (1973) observed that this activity initially increased but subsequently decreased with continuing growth of the tumour. However, the absolute values for the whole spleen remained elevated during the course of tumour bearing. This study was carried out using the gross population of the spleen cells and therefore cannot be taken as a measure of the splenic lymphocyte DNA synthesis. These workers also reported a marked increase of cells in the spleen responding to a variety of non-tumour antigens and also that the splenic haematopoietic cells capable of colony formation on adoptive transfer were observed to be greatly increased during tumour bearing. Whilst these observations are not contrary to those reported here, our findings may gain strong support from the observations of Vanky *et al.* (1973) who showed that in mixed lymphocyte–target cell interaction tests, LNC draining large tumours of long duration in human cancer, failed to respond to the autochthonous tumour cells but retained their ability to react to allogeneic lymphocytes or to phytohaemmaglutinin, indicating the specificity of non-responsiveness.

We have shown that the host's LNC response to the tumour becomes depressed soon after the early period of tumour growth. This may find an interesting correlation in the control of the delayed hypersensitivity reaction in mice painted with certain skin sensitizing agents (Asherson and Barnes, 1973; Zembala *et al.*, 1975). It has been shown that pretreatment of mice with the picrylating agent picryl sulphonic acid depresses or abolishes the DNA synthetic response to the same agent as well as to picryl chloride in the lymph nodes, but not in the spleen. Delayed hypersensitivity, but not the antibody response, is markedly affected, reflecting on the composition of these 2 types of lymphoid organs—the lymph nodes consisting predominantly of " T " cells while the spleen containing nearly equal proportions of both " T " and " B " cells (Raff, 1971). Although the DNA synthetic response in the LNC of the tumour bearing mice studied here may not undergo such dramatic changes, it is nevertheless possible that a similar basis may underlie both these phenomena. It would therefore be interesting to see if such " suppressor factor(s) " could be demonstrable in the tumour hosts and if so to identify and study their immunobiochemical nature.

We wish to thank Miss D. Murray and Mr R. Clay for their valuable technical assistance.

The continued support of the North West Cancer Research Fund is gratefully acknowledged.

### REFERENCES

ALEXANDER, P., BENSTEAD, J., DELORME, E. J. & HODGETT, J. (1969) The Cellular Immune Response to Primary Sarcomata in Rats. II. Abnormal Response of Nodes Draining the Tumour. *Proc. R. Soc. B*, **174**, 237.

ASHERSON, G. L. & BARNES, R. M. R. (1973) Contact Sensitivity in the Mouse: XII. The Use of DNA Synthesis *in vivo* to Determine the Anatomical Location of Immunological Unresponsiveness to Picryl Chloride. *Immunology*, **25**, 495.

BALDWIN, R. W., PRICE, M. R. & ROBSINS, R. A. (1972) Blocking of Lymphocyte Mediated Cytotoxicity for Rat Hepatoma Cells by Tumour-specific Antigen–antibody Complexes. *Nature, New Biol.*, **238**, 185.

CHANDRADASA, K. D. (1973) Development and Specific Suppression of Concomitant Immunity in two Syngeneic Tumour-host Systems. *Int. J. Cancer*, **11**, 648.

DECKERS, P. J., DAVIS, R. C., PARKER, G. A. & MANNICK, J. A. (1973) The Effect of Tumor Size on Concomitant Tumor Immunity. *Cancer Res.*, **33**, 33.

EDWARDS, A. J., SUMNER, G. F., ROWLAND, G. F. & HUND, C. M. (1971) Changes in Lymphoreticular Tissues during Growth of Murine Adenocarcinoma. I. Histology and Weights of Lymph Nodes, Spleen and Thymus. *J. natn. Cancer Inst.*, **47**, 301.

EVANS, C. A., GORMAN, L. R., ITO, Y. & WEISER, R. S. (1962) Antitumour Immunity in Shope Papilloma Carcinoma Complex of Rabbits. I. Papilloma Regression Induced by Homologous and Autologous Tissue Vaccines. *J. natn. Cancer Inst.*, **29**, 277.

HADDOW, A. (1965) Immunology of the Cancer Cell. Tumour Specific Antigens. *Br. med. Bull.*, **21**, 133.

HADDOW, A. & ALEXANDER, P. (1964) An Immunological Method of Increasing the Sensitivity of Primary Sarcomas to Local Irradiation with X-rays. *Lancet*, i, 452.

HELLSTRÖM, I. & HELLSTRÖM, K. E. (1969) Studies on the Cellular Immunity and its Serum Mediated Inhibition by Moloney-virus Induced Mouse Sarcoma. *Int. J. Cancer*, **4**, 587.

HELLSTRÖM, I., HELLSTRÖM, K. E., EVANS, C. A., HEPPNER, G. H., PIERCE, G. E. & YOUNGE, J. P. S. (1969) Serum Mediated Protection of Neoplastic Cells from Inhibition by Lymphocytes Immune to the Tumour Specific Antigens. *Proc. natn. Acad. Sci. U.S.A.*, **62**, 362.

KONDA, S., NAKAO, I. & SMITH, R. T. (1973) The Stimulatory Effect of Tumor Bearing upon the T and B Cell Subpopulations of the Mouse Spleen. *Cancer Res.*, **33**, 2247.

RAFF, M. C. (1971) Surface Antigen Markers for Distinguishing T and B Lymphocytes in Mice. *Transplantn Rev.*, **6**, 52.

ROSENAU, W. & MOON, H. D. (1966) Cellular Reaction to Methylcholanthrene-induced Sarcomas Transplanted to Isogeneic Mice. *Lab. Invest.*, **15**, 1212.

SJÖGREN, H. O., HELLSTRÖM, I., BANSAL, S. C. & HELLSTRÖM, K. E. (1971) Suggestive Evidence that the " Blocking Antibodies " of Tumour Bearing Individuals may be Antigen Antibody Complexes. *Proc. natn. Acad. Sci. U.S.A.*, **68**, 1372.

VAAGE, J. (1972) Specific Densensitization of Resistance against a Syngeneic Methylcholanthrene-induced Sarcoma in C3 Hf Mice. *Cancer Res.*, **32**, 193.

VAAGE, J. (1973) Influence of Tumor Antigen on Maintenance *versus* Depression of Tumour-specific Immunity. *Cancer Res.*, **33**, 493.

VANKY, F., STJERNSWARD, J., NILSONNE, U. & SUNDBLAD, R. (1973) Differences in the Tumor-associated Reactivity of Blood Lymphocytes and Tumor-draining Lymph Node Cells in Sarcoma Patients. *J. natn. Cancer Inst.*, **51**, 17.

WHITNEY, R. B., LEVY, J. G. & SMITH, A. G. (1974) Influence of Tumor Size and Surgical Resection on Cell-mediated Immunity in Mice. *J. natn. Cancer Inst.*, **53**, 111.

ZEMBALA, M., ASHERSON, G. L., MAYHEW, B. & KREJCI, J. (1975) *In vitro* Absorption and Molecular Weight of Specific T-cell Suppressor Factor. *Nature, Lond.*, **253**, 72.